# Flipping the Classroom in Otolaryngology Residencies

**DOI:** 10.7759/cureus.8981

**Published:** 2020-07-03

**Authors:** William J Kohler, Nicole M Favre, Daniel C O'Brien, Michele M Carr

**Affiliations:** 1 Biomedical Sciences, West Virginia School of Osteopathic Medicine, Lewisburg, USA; 2 Otolaryngology, Jacobs School of Medicine and Biomedical Sciences, University at Buffalo, Buffalo, USA; 3 Otolaryngology-Head & Neck Surgery/Rhinology, University of Alberta, Edmonton, CAN

**Keywords:** otolaryngology residency, otolaryngology training, medical education, flipped classroom, graduate medical education, curricular methods, program directors

## Abstract

Objective

To understand the use of the flipped classroom (FC) - learning core content prior to an academic session, with class time devoted to applying this content - in otolaryngology residency education.

Methods

An electronic survey of 107 otolaryngology program directors (PDs), including demographic details, the flipped classroom perception instrument (FCPI), and the otolaryngology programs' current use of FC.

Results

Forty-four (41%) PDs completed the FCPI. Seventy-one point one (71.1%) of respondents were male, 60% were 30-49 years, and the remainder were older. Sixty-two percent (62%) had fellowships associated with their program, 21.7% of programs used the FC model Very Often, 17.4% Somewhat Often, 28.3% Sometimes, 17.4% Somewhat Rarely, 8.7% Very Rarely, and 6.5% Never.

Attitudes toward FC principles were positive with modes “strongly agree” for all, except for “online modules enhance learning” where the mode was “slightly agree” with significantly higher scores for PDs over age 50 than for those younger (4.17 vs. 3.63, p=0.033). There were no other significant differences comparing male vs. female PDs, younger vs. older PDs, smaller vs. larger programs, programs with or without fellowships, programs with 100% vs. <100% board exam pass rates, or programs in different geographical regions. The pre-class activity mean score was 4.34 (95% CI 4.12-4.56) and the in-class mean score was 4.18 (95% CI 3.99-4.37). There was no significant correlation between the likelihood of using a flipped classroom and attitude scores.

Conclusion

PDs value both the pre-class and interactive in-class principles of FCs but only 37.8% of programs use FC often, suggesting that practical approaches to implementation in this group could improve education in this population.

## Introduction

Traditionally, educational content is delivered in graduate medical education (GME) through expert lectures; however, resident workload limitations have presented a challenge to presenting a robust curriculum [1-2]. With the advent of online learning, a concept known as the flipped classroom (FC) has emerged. This consists of the delivery of educational content to students prior to the in-class activity, thus allowing the class itself to be devoted to the application of the material [3]. A common model is the distribution of online videotaped lectures prior to scheduled didactic sessions. The concept originated in undergraduate institutions and has been reported to have been used with success at the high school, undergraduate, and medical school levels, with respect to class attendance and participation [3-4]. Recent attempts to translate the FC model in a residency setting have seen success in pharmacy, internal medicine, and emergency medicine [2-3]. In an attempt to quantify the perceptions of this new model, the flipped classroom perception instrument (FCPI) was designed and validated with a traditional didactic-based internal medicine residency curriculum, yielding overall internal consistency reliability of 0.84 [3]. The aim of this investigation is to determine program director (PD) attitudes toward the utilization of FCs in otolaryngology residency education using the FCPI.

## Materials and methods

PDs from every allopathic otolaryngology residency program in the United States (n=107) were sent an email inviting them to participate in a brief, anonymous survey. The survey questionnaire was created based on the FCPI, a measure of individual opinions of the model developed iteratively in the literature [5]. Study data were collected and managed using Research Electronic Data Capture (REDCap) hosted at West Virginia University [6-7]. REDCap is a secure, web-based platform designed to support data capture for research studies. Individuals were only able to complete the assessment once, and their responses were kept anonymous and confidential. The survey was sent out four times between November 2017 and January 2018. It included a seven-item Likert scale questionnaire, consisting of questions concerning three pre-class activities and four in-class activities. The pre-class activities included attitudes toward online modules and toward learning key content prior to class, while in-class activities measured attitudes toward in-class discussion, application, interaction, and team projects. PDs were also asked to provide basic demographic information, including age, gender, and time spent as a program director. Program parameters included the number of residents admitted yearly to the program, the number of full-time faculty present, the presence of fellows in the program, and geographic location in the United States.

This protocol was approved by the West Virginia University Institutional Review Board. Statistical evaluation was performed using IBM SPSS Statistics (Version 25.0, Armonk, NY: IBM Corp 2017).

## Results

Of the 107 program directors determined to have met the survey criteria, 48 (45%) responded to the survey and 44 (41%) completed the FCPI. The demographics of the participating otolaryngology residency program director group are illustrated in Table 1.

**Table 1:** Program director (PD) demographics

	Response	N	%
	Age of PD (years)		
	30-49	22	60.0
	50 or older	17	40.0
	Gender		
	Male	27	71.1
	Female	12	28.9
	Time as PD		
	Less than 2 years	12	30.4
	Greater than 2 but less than 5	8	21.7
	Greater than 5 but less than 10	12	28.3
	More than 10 years	8	19.6

A majority of otolaryngology PDs who responded were males (71%), with most identifying themselves to be in the 30-49-year age group (60%). Table 2 summarizes the demographics of each of the residency programs included in the survey.

**Table 2:** Program demographics

Response	N	%
Number of residents admitted per year		
2 or fewer	10	24.4
2.5*	2	4.9
3	14	34.1
4	7	17.1
5	5	12.2
Other	3	7.3
Number of full-time faculty in program		
5 or fewer	1	2.4
6-10	8	19.5
11-15	11	26.8
16-20	4	9.8
21 or more	17	41.5
Region of United States		
Midwest	12	29.8
Northeast	14	34.0
Southeast	8	19.1
Southwest	2	4.3
West	5	12.8

Wide variation was observed in the number of residents admitted per year and the number of full-time faculty, with representation from each geographical region of the United States. Out of all of the programs, 62% had fellowships and 70% had a 100% American Board of Otolaryngology graduate resident pass rate over the last five years.

The PDs’ individual perceptions of various aspects of the flipped classroom model are illustrated in Table 3.

**Table 3:** Program responses regarding flipped classroom

	Never n (%)	Very Rarely n (%)	Somewhat Rarely n (%)	Sometimes n (%)	Somewhat Often n (%)	Very Often n (%)
Have utilized a flipped classroom in this residency program	3 (6.5)	4 (8.7)	8 (17.4)	13(28.3)	8 (17.4)	10 (21.7)
Question stem: Please indicate how much you agree with the following statements, considered in the context of residency training in your program						
Means of enhancing learning		Strongly Disagree (%)	Disagree (%)	Neutral (%)	Agree (%)	Strongly Agree (%)
Pre-class						
Online modules enhance learning		0	2	33	42	22
Learning key content prior to class sessions enhance learning		2	0	4	22	71
Combination of online modules with in-class application enhance learning		0	2	27	31	40
In-class						
Interactive applied in-class activities enhance learning		2	0	7	29	62
In-class application of core content enhances learning		2	0	4	33	60
Discussion of core content		2	0	7	27	64
Team projects enhance learning		2	7	24	31	36

Regarding the frequency of flipped classroom use in their programs, 10 (21.7%) otolaryngology PDs replied “Very Often”, 8 (17.4%) replied “Somewhat Often”, 13 (28.3%) replied “Sometimes”, 8 (17.4%) replied “Somewhat Rarely”, 4 (8.7%) replied “Very Rarely”, and 3 (6.5%) replied “Never”. Of the seven statements on the FCPI, PDs most frequently agreed that “Learning key content prior to class sessions enhances learning” (71%), while “Team projects enhance learning” (36%) was the most frequently disputed response. Attitudes toward FC principles were positive with modes “Strongly Agree” for all, except for “online modules enhance learning” where the mode was “Slightly Agree,” with significantly higher scores for PDs over age 50 than for those younger (4.17 vs. 3.63, p=0.033). There were no other significant differences for these principles when comparing male vs. female PDs, younger vs. older PDs, smaller (less than three residents per year) vs. larger (three or more residents per year) programs, programs with or without fellowships, programs with 100% vs. <100% American Board of Otolaryngology exam pass rates, or programs in different geographical regions. Pre-class activity mean score was 4.34 (95% CI 4.12-4.56) and the in-class mean score was 4.18 (95% CI 3.99-4.37). There was no significant correlation between the likelihood of using a flipped classroom and mean attitude scores.

## Discussion

With limitations to resident work hours and the need to balance time spent studying didactic material with patient care, the FC model may present a unique solution to maximize the effectiveness of instructional time. In an ideal medical education scenario presented by Prober and Khan, medical education is divided into three phases: building a framework of core knowledge, embedding knowledge through interactive formats, and encouraging the in-depth processing of specific knowledge [8]. The framework stage consists of the use of readily available materials for self-study, which could include written material, videos, or other electronic presentations. This is followed by an application of the previously learned material in the interactive stage such as in case discussions or problem-solving [8]. This approach is applicable in the realm of graduate medical education. Critics suggest that the differing nature of undergraduate and graduate medical education may limit the value of FC for residents. Due to emergencies relating to patient care and other clinical commitments, residents are not able to be continually invested in mastering the curriculum, making them less likely to engage with the pre-class material [1]. Preparation before an academic meeting may not be prioritized and thus not occur, as Young et al. found in their study with emergency medicine residents [2]. Their group ran two FC trials: in the first, only 64% of residents watched the video ordered before the teaching session, but in the second trial, 85% had done so [2]. In contrast, Burns suggests that an FC model may be ideal when limited duty hours discourage resident attendance at scheduled didactic sessions [9]. From a teaching clinician perspective, the FC model is attractive; clinicians may prefer to use already available online teaching resources, or record their own, with a better chance that every resident in their program will be able to use it at some point - not missing educational opportunities due to vacation, illness, duty-hour restrictions, or fatigue. Another opportunity with this model is the possibility of developing national, collaborative teaching resources such as the online free EKG interpretation course developed for Emergency Medicine residents and described by Burns et al. [10]. As well, with the ACGME Milestones program [11], academic clinicians need to spend better quality educational time with trainees in order to evaluate them on various parameters of their medical knowledge. This is more easily done in a case discussion or problem-solving session than in typical lecture situations.

FC usage has been demonstrated to improve long-term retention of information in residents [12]. In a cohort of PGY-3 pediatric emergency medicine residents, an FC model combined with interactive application-style questions led to improved scores on core pediatric emergency medicine topics [12]. Blair et al. did pre-tests and post-tests followed by a six-month follow-up test and found an improvement after their FC trial in internal medicine residents; however, there was no control group [13]. In similar fashion to the FC model, supplemental videos made available to otolaryngology residents but not scheduled in the curriculum and not made part of an FC paradigm led to improved scores on junior residents’ otolaryngology in-training examinations in the pediatric otolaryngology, otology, and facial plastic surgery sections [14]. Otolaryngology residents have access to numerous on-line educational videos through the American Academy of Otolaryngology-Head and Neck Surgery [15] and those created by new multi-institution collaboratives [16-17], which may form the base for an FC program in this specialty.

In addition to improved empirical measures of academic performance, first-year obstetrics and gynecology residents who had taken an elective program in their fourth year reported the highest satisfaction in the FC portion of the course, to which they also attributed their improved clinical confidence as compared to the traditional didactic portion [18]. Program interest similarly increased in a surgery core clerkship, with students participating in FC sessions indicating higher career interest and learner satisfaction, although with identical standardized test performance [19]. Our survey results corroborate with the overall positive perception of FC implementation previously seen in the literature. However, the finding of older program directors more strongly supporting the FC model contrasts with previous literature, in which younger female PDs of internal medicine residency programs were more likely to perceive FC as favorable [3]. Cooper et al. noted that at the time of their work, measures of FCs were based on satisfaction alone, and studies had failed to address the feasibility of modifying the existing curriculum [1]. Recent meta-analyses note heterogeneity, high risk of bias, and lack of rigor in studies on FC, making it difficult to draw conclusions about its academic value [19-20]. Perhaps examining contributions of budget, resources, and receptive faculty in future studies could lead to increased accessibility and improved PD willingness to flip the classroom in otolaryngology.

The limitations of our study include the small sample size, although this response rate is typical of otolaryngology PDs in recent publications and in our experience [21]. We surveyed only PDs and may have found different results if residents or other faculty were queried. The questionnaire we used examined attitudes only, so barriers to the institution of this curricular method were not evaluated.

## Conclusions

This study highlights the attitudes, perceptions, and use of the FC model among allopathic otolaryngology residency PDs, which were determined using the FCPI. Roughly two-thirds of PDs indicated that they had used the FC model at least sometimes, with a majority supporting the principles of enhanced pre- and in-class learning associated with the model. Despite having a positive outlook on the components of the FC and having very limited curricular time in the entire otolaryngology residency, FC is not universally employed by these educators. Future work should be directed toward maneuvers that may facilitate the incorporation of FC in otolaryngology residency.
